# The pathogen spectrum of influenza-like illness in Guilin, China, 2023–2024

**DOI:** 10.3389/fpubh.2026.1788314

**Published:** 2026-03-18

**Authors:** Jin Cao, Hu Long, Wenyan Tian, Yingli Qu, Guoyong Mei, Haijun Du, Zhiqiang Xia, Mi Liu, Qinqin Song, Ru Cai, Jun Han

**Affiliations:** 1 The First Affiliated Hospital of Anhui University of Science and Technology (Huainan First People's Hospital), Huainan, China; 2 National Key Laboratory of Intelligent Tracking and Forecasting for Infectious Diseases, National Institute for Viral Disease Control and Prevention, Chinese Center for Disease Control and Prevention, Beijing, China; 3 School of Medicine, Anhui University of Science and Technology, Huainan, China; 4 Guilin Center for Disease Control and Prevention, Guilin, China

**Keywords:** acute respiratory infection, co-infection, epidemiological characteristics, influenza virus, influenza-like illness

## Abstract

**Objective:**

To investigate the pathogen spectrum and epidemiological characteristics of influenza-like illness (ILI) in Guilin, China, from 2023 to 2024.

**Methods:**

Between October 2023 and October 2024, 400 pharyngeal swabs were collected weekly from ILI patients and 18 respiratory pathogens were detected using quantitative real-time PCR (qPCR). Pathogen spectrum and epidemiological characteristics were analyzed.

**Results:**

Of the 400 samples, the overall pathogen-positive rate was 74.0% (296/400). Among the detected pathogens, Influenza (Flu) was the main pathogen (44.0%, 176/400), followed by SARS-CoV-2 (12.3%, 49/400) and *Mycoplasma pneumoniae* (MP) (8.8%, 35/400). The detection rate is highest in the age group of 45–59 years old, reaching 91.7%. The detection rates for Flu, SARS-CoV-2, MP, HAdV, RSV, EV, and HPIV showed significant differences among age groups (*p* < 0.05). The peak period of positive detection rate was autumn and winter (Nov 2023–Mar 2024). Flu (H3N2 and IBV) dominated in autumn and winter, but was gradually replaced by SARS-CoV-2 and MP thereafter. By autumn 2024, SARS-CoV-2 and HRV were predominant. Co-infections were identified in 14.2% (42/296) of positive cases, with the combination of Flu and MP being the most common (19.0%).

**Conclusion:**

During the study period, ILI in Guilin was primarily driven by Flu, which co-circulated with other pathogens including SARS-CoV-2 and MP, demonstrating distinct seasonal patterns and demographic distributions.

## 1 Introduction

Acute respiratory infections (ARIs) are highly prevalent infectious diseases worldwide, characterized by fast transmission, strong seasonality, and high population susceptibility (1, 2). The disease burden of ARIs is particularly prominent among vulnerable populations, including children, the older adults, pregnant women, obesity, and individuals with underlying comorbidities (3). ARIs are clinical acute diseases caused by pathogens with acute exacerbation of respiratory symptoms (usually lasting no more than 21 days). The main symptoms include cough, sputum production, shortness of breath, sore throat, and runny nose (4). ARIs mainly includes acute upper respiratory tract infections, acute bronchitis, and community acquired pneumonia (CAP), with common pathogenic microorganisms being viruses and bacteria (including atypical pathogens). In ARIs, upper respiratory tract infection accounts for 70–90%, of which 70–80% are caused by viruses. ARIs accounted for a striking mortality rate of up to 83% in low- and middle-income countries (5). The etiology of ARIs is complex, encompassing a wide spectrum of viral, bacterial, and atypical pathogens. Notably, viruses are implicated in approximately 60–80% of all cases (6, 7). The clinical presentation of ARIs is highly variable, ranging from self-limiting influenza-like illness (ILI) to severe acute respiratory infection and fatal outcomes, especially for vulnerable populations (3).

ILI constitutes a specific and substantial subset of ARIs, accounting for approximately 62% of all cases (8). The World Health Organization (WHO) defines ILI as an acute presentation of fever (temperature ≥38 °C), accompanied by cough or sore throat, in the absence of an alternative clinical diagnosis (9). The most common viral pathogens responsible for ILI include influenza virus, adenovirus, rhinovirus, and respiratory syncytial virus and others (10). Surveillance of ILI is a cornerstone of public health preparedness. The persistent threat of emerging infectious diseases to global health security further underscores the value of ILI surveillance, which can function as an early warning system for outbreaks (11). Notably, during the COVID-19 pandemic, existing ILI surveillance frameworks played an indispensable role in initial case detection and tracking the spread of SARS-CoV-2 (12).

Concurrently, a notable increase in ILI cases was observed in October 2023, coinciding with reported influenza outbreaks across multiple regions in China. To elucidate the pathogen spectrum of ILI in Guilin, this study collected 400 pharyngeal swab samples from ILI patients in Guilin, Guangxi Zhuang Autonomous Region, China, between October 2023 and October 2024. Eighteen common respiratory pathogens were detected to define the local pathogen spectrum of ILI for enhancing regional prevention and control strategies against ARIs.

## 2 Materials and methods

### 2.1 Sample

A total of 400 pharyngeal swab samples were collected between October 2023 and October 2024 from outpatients presenting with influenza-like illness (ILI) at the fever clinics of Hospital. The ILI case definition included axillary temperature >38 °C accompanied by cough or sore throat. Pharyngeal swabs were obtained within 24 h of symptom onset. Sampling was conducted based on the weekly number of ILI consultations: when the weekly count exceeded 20 cases, 20 samples were collected; otherwise, all eligible cases were enrolled. All swabs were immediately stored at −80 °C upon collection and subsequently transported via cold chain to the laboratory for pathogen detection. Demographic information (gender, age) and sample collection date were recorded for each patient.

### 2.2 Sample processing and nucleic acid extraction

Following thawing at 4 °C, all pharyngeal swab samples were aseptically aliquoted into cryovials. Two hundred microliter of each sample was used for nucleic acid extraction through a commercial nucleic acid extraction kit (Xi’an Tianlong Science and Technology Co., Ltd.) using an automated extraction system (NP968, Xi’an Tianlong Science and Technology Co., Ltd.) according to the manufacturer’s protocol. The extracted nucleic acids were stored at −80 °C and subjected to pathogen detection within 48 h.

### 2.3 Pathogen detection

A comprehensive detection for 18 common respiratory pathogens was performed, including 14 RNA viruses [influenza A virus (IAV), influenza B virus (IBV), human rhinovirus (HRV), human parainfluenza virus types 1–3 (HPIV1–3), human coronaviruses (HCoV-OC43, HCoV-NL63, HCoV-229E, HCoV-HKU1), respiratory syncytial virus (RSV), human metapneumovirus (HMPV), enterovirus (EV), and SARS-CoV-2], two DNA viruses [human adenovirus (HAdV) and human bocavirus (HBoV)], as well as two atypical bacterial pathogens [*Mycoplasma pneumoniae* (MP) and *Chlamydia pneumoniae* (CP)]. Nucleic acid amplification was conducted in accordance with the manufacturer’s protocol using the GoldStar Probe One-Step RT-qPCR kit (Jiangsu Kangwei Century Bio-tech Co., Ltd., China). Results were interpreted based on the cycle threshold (Ct) value (<38) and amplification curves.

### 2.4 Grouping criteria and definitions

Study cases were divided into four age groups across the entire age spectrum: children (<18 years, *n* = 142), young adults (18–44 years, *n* = 219), middle-aged adults (45–59 years, *n* = 25), and older adults (≥60 years, *n* = 15). The pediatric group (<18 years) was further subdivided into: infants (<1 year, *n* = 9), toddlers (1–3 years, *n* = 28), preschoolers (4–6 years, *n* = 35), school-age children (7–12 years, *n* = 28), and adolescents (13–17 years, *n* = 42).

Seasonal analysis was conducted based on the following definitions: Spring (March–May), Summer (June–August), Autumn (September–November), and Winter (from December 2023 to February 2024).

### 2.5 Statistical analysis

Data management was performed using Microsoft Excel 2010. All statistical analyses were conducted with SPSS software (version 27.0), and GraphPad Prism was employed for graphs generation and refinement. The detection rates of respiratory pathogens were compared across different genders, age groups, months, and seasons. Categorical data were presented as numbers and percentages (n, %). Intergroup comparisons were assessed using the chi-square test (χ^2^). A two-sided *p*-value < 0.05 was considered statistically significant. When more than 20% of expected cell frequencies were <5, Fisher’s exact test was applied as an alternative. Following significant chi-square results for monthly pathogen detection rates, *post hoc* comparisons were performed using Bonferroni-corrected standardized residuals to identify statistically homogeneous subsets; groups sharing the same subscript letter do not differ significantly at α = 0.05.

## 3 Results

### 3.1 Pathogen detection

**Figure 1:**
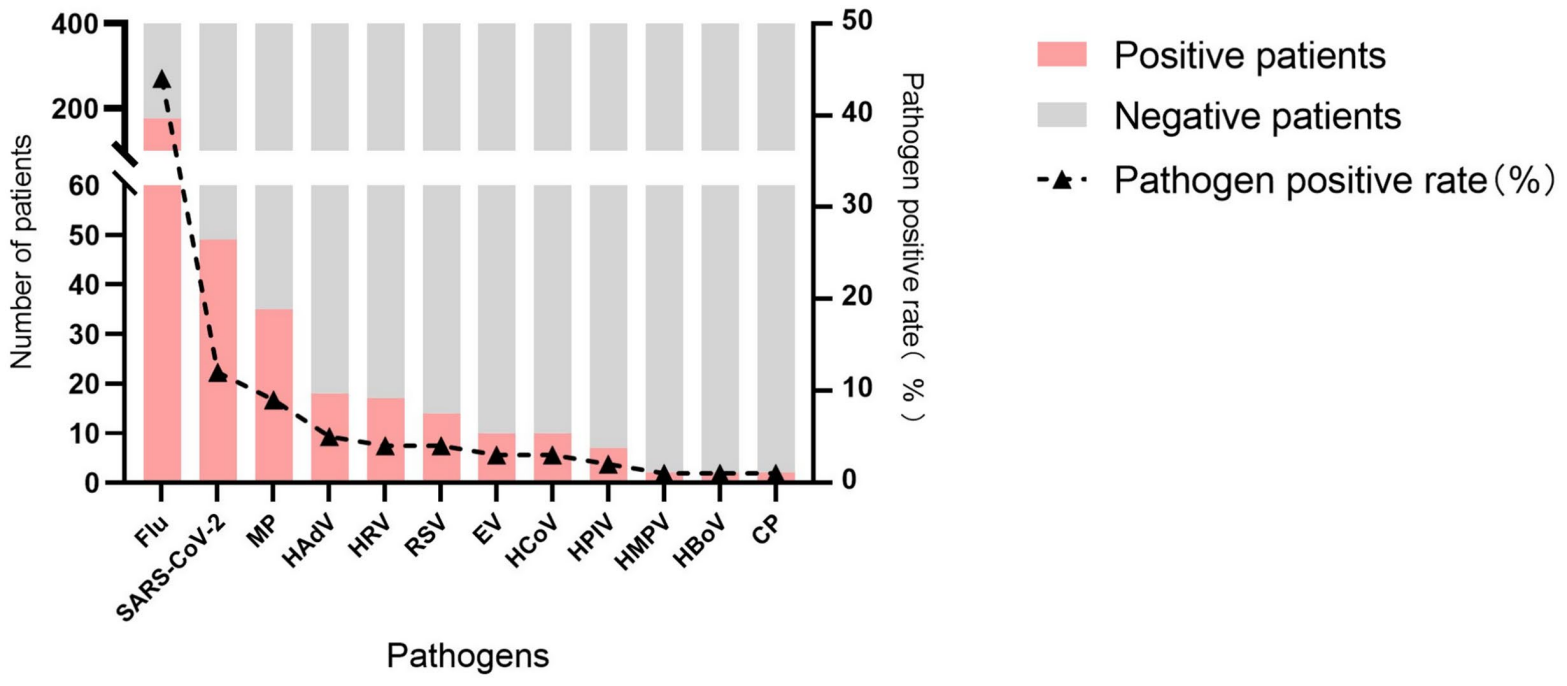
Detection of respiratory pathogens in Guilin, China, from October 2023 to October 2024.

A total of 400 pharyngeal swab samples were collected from ILI patients in Guilin between October 2023 and October 2024. RT-PCR testing showed that at least one respiratory pathogen was detected in 296 samples, resulting in an overall positivity rate of 74.0% (296/400). Flu was the most predominant pathogen detected in 44.0% (176/400) of the samples, followed by SARS-CoV-2 (12.3%, 49/400) and MP (8.8%, 35/400). The other pathogens were detected at the following rates, in descending order: HAdV (4.5%, 18/400), HRV (4.3%, 17/400), RSV (3.5%, 14/400), EV (2.5%, 10/400), HCoV (2.5%, 10/400), HPIV (1.8%, 7/400), HMPV (0.5%, 2/400), HBoV (0.5%, 2/400), and CP (0.5%, 2/400) (Figure 1).

### 3.2 Demographic characteristics

**Figure 2:**
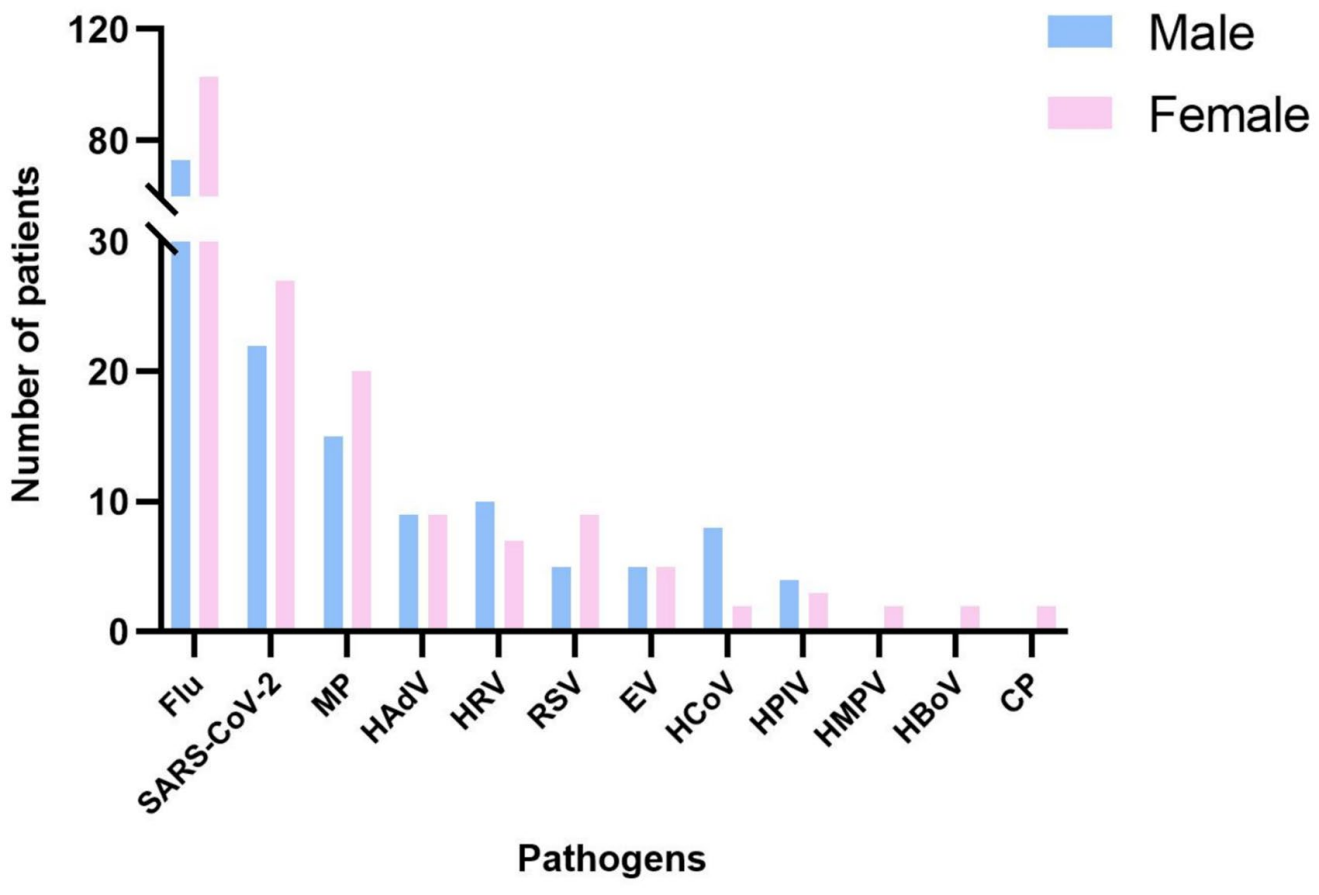
Detection of respiratory pathogens by gender in Guilin, China, from October 2023 to October 2024.

Among the 400 enrolled cases, 187 were male and 213 were female, yielding a male-to-female ratio of approximately 1:1.4. The pathogen detection rate was 70.1% (131/187) in males and 77.5% (165/213) in females. However, the overall difference in pathogen detection rate between genders was not statistically significant (*p* > 0.05) (Figure 2; Supplementary Table S1).

**Figure 3:**
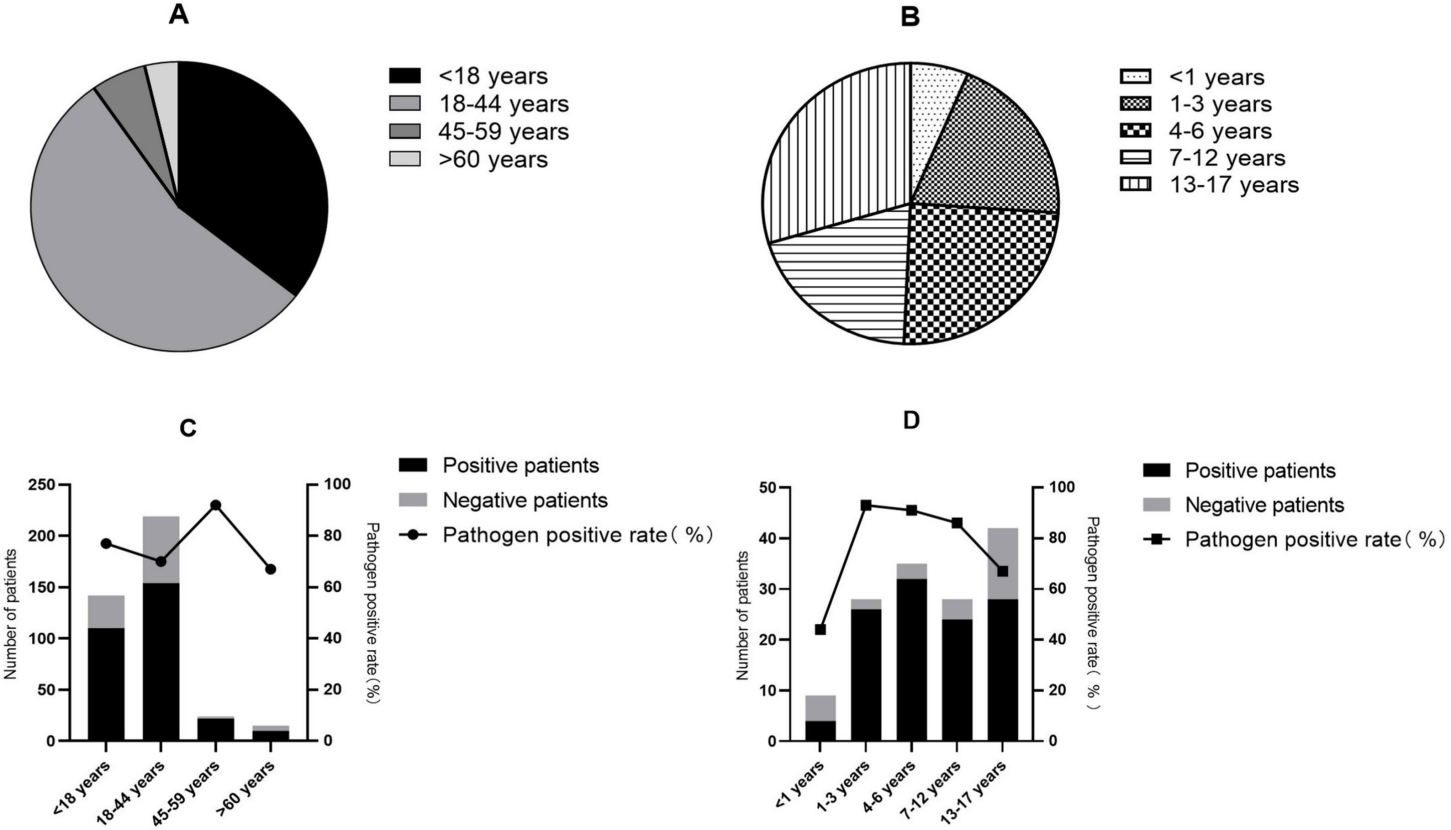
The composition of pathogen detection in different age group. **(A)** Age distribution of the total patients (*N* = 400). **(B)** Age distribution of the pediatric patients (participants aged <18 years, *n* = 142). **(C)** Overall pathogen detection rate across all age groups. **(D)** Pathogen detection rate in different age-group of pediatric patients.

**Figure 4:**
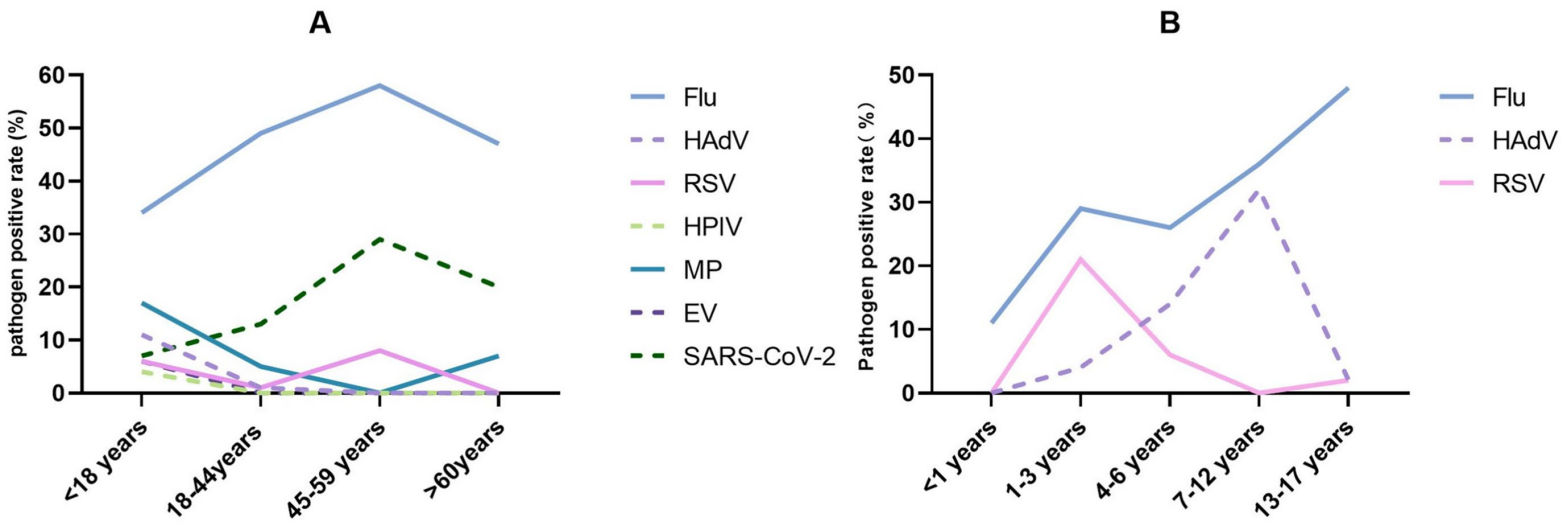
Age-specific detection patterns of major respiratory pathogens (pathogens with low detection rates or no clear age-related trends are not displayed): **(A)** Total group and **(B)** pediatric subgroup.

The 400 cases in this study had a median age of 20 years (interquartile range: 13–29 years) and were divided into four age groups (<18, 18–44, 45–59, ≥60 years; Figure 3A). The overall pathogen detection rate was highest in the 45–59 years group (91.7%, 22/24) and lowest in the ≥60 years group (66.7%, 10/15) (Figure 3C; Supplementary Table S2). Significant age-specific variations were observed for Flu (*p* = 0.017), HAdV (*p* < 0.001), RSV (*p* = 0.046), HPIV (*p* = 0.037), MP (*p* < 0.001), EV (*p* = 0.011), and SARS-CoV-2 (*p* = 0.028), respectively (Table 1; Figure 4). The Flu detection rate was highest in adults aged 45–59 years (58.3%) and lower in children (33.8%). In contrast, HAdV, MP, and EV were almost exclusively detected in the <18 years group (HAdV: 11.3%, MP: 16.9%, EV: 6.3%). RSV showed a bimodal pattern, with peaks in children (6.3%) and adults aged 45–59 years (8.3%). The detection rates of SARS-CoV-2 increased with age, rising from 7.0% in children to 29.2% in adults aged 45–59 years.

**Table 1:** Pathogen-positive counts by age group in total and pediatric subgroups.

	Total group (*N* = 400)						Pediatric subgroup (*n* = 142)					
Age group (years), *n* (%)	<18	18–44	45–59	>60	χ^2^	*P*-value	<1	1–3	4–6	7–12	13–17	*P*-value
Flu*	48 (33.8)	107 (48.9)	**14 (58.3)**	7 (46.7)	10.135	**0.017**	1 (11.1)	8 (28.6)	9 (25.7)	10 (35.7)	20 (47.6)	0.169
HAdV	**16 (11.3)**	2 (0.9)	0 (0.0)	0 (0.0)		**<0.001**	0 (0.0)	1 (3.6)	5 (14.3)	**9 (32.1)**	1 (2.4)	**0.003**
HRV	9 (6.3)	8 (3.7)	0 (0.0)	0 (0.0)		0.221	0 (0.0)	2 (7.1)	4 (11.4)	1 (3.6)	2 (4.8)	0.545
HCoV*	4 (2.8)	5 (2.3)	1 (4.2)	0 (0.0)		0.761	0 (0.0)	2 (7.1)	1 (2.9)	0 (0.0)	1 (2.4)	0.634
RSV	**9 (6.3)**	3 (1.4)	**2 (8.3)**	0 (0.0)		**0.046**	0 (0.0)	**6 (21.4)**	2 (5.7)	0 (0.0)	1 (2.4)	**0.009**
HMPV	2 (1.4)	0 (0.0)	0 (0.0)	0 (0.0)		0.338	0 (0.0)	2 (7.1)	0 (0.0)	0 (0.0)	0 (0.0)	0.292
HPIV*	**6 (4.2)**	1 (0.5)	0 (0.0)	0 (0.0)		**0.037**	0 (0.0)	1 (3.6)	0 (0.0)	0 (0.0)	0 (0.0)	0.654
HBoV	1 (0.7)	1 (0.5)	0 (0.0)	0 (0.0)		0.889	0 (0.0)	0 (0.0)	0 (0.0)	1 (3.6)	0 (0.0)	0.638
CP	1 (0.7)	1 (0.5)	0 (0.0)	0 (0.0)		0.889	1 (11.1)	2 (7.1)	9 (25.7)	7 (25.0)	5 (11.9)	0.216
MP	**24 (16.9)**	10 (4.6)	0 (0.0)	1 (6.7)		**<0.001**	0 (0.0)	2 (7.1)	3 (8.6)	1 (3.6)	0 (0.0)	0.191
EV	**9 (6.3)**	1 (0.5)	0 (0.0)	0 (0.0)		**0.011**	0 (0.0)	2 (7.1)	5 (14.3)	1 (3.6)	1 (2.4)	0.298
SARS-CoV-2	10 (7.0)	29 (13.2)	**7 (29.2)**	3 (20.0)		**0.028**	2 (22.2)	2 (7.1)	2 (5.7)	2 (7.1)	2 (4.8)	0.762

(1) Data are presented as *n* (%). *P*-values were calculated by the Chi-square test (or Fisher’s exact test, if applicable). *P* < 0.05 was considered statistically significant and is highlighted in bold. (2) * Flu detection is presented here as a combined category (including H1N1, H3N2, and IBV) and is analyzed separately in subsequent sections. Due to low individual counts, HCoV-OC43, HCoV-NL63, HCoV-229E, and HCoV-HKU1 are reported as a combined group (HCoV), and HPIV1–3 are reported collectively as HPIV.

The pediatric subgroups were divided into five age groups (<1, 1–3, 4–6, 7–12, and 13–17 years; Figure 3B), and the detection rate varied markedly among them, from 44.4% in infants (<1 year) to 91.4% in preschoolers (4–6 years) (*p* = 0.015; Figure 3D; Supplementary Table S2). The detection rate of Flu showed an increasing trend with age, rising from 11.1% in infants (<1 year) to 47.6% in adolescents (13–17 years). HAdV infection displayed a clear school-age predominance, with the highest detection rate observed in the 7–12 years subgroup (32.1%). RSV mainly affected infants and young children, peaking in the 1–3 years subgroup (21.4%). The differences across subgroups were statistically significant for both HAdV and RSV (*p* < 0.05) (Figure 4).

**Figure 5:**
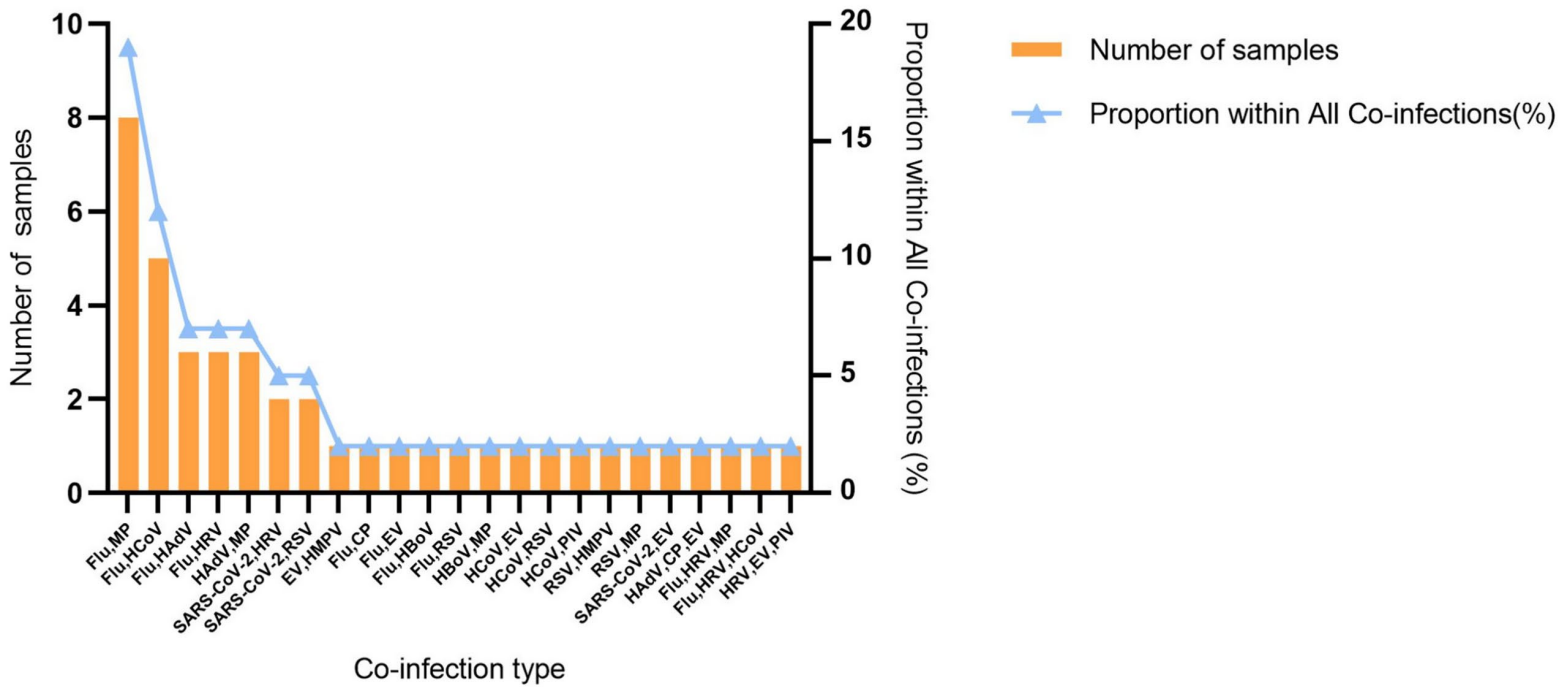
Profile of pathogen co-infections.

Among the 296 pathogen-positive cases, 254 (85.8%) had single infections, and 42 cases (14.2%) were co-infections. The most common co-infection pattern was both Flu and MP (19.0%, 8/42), followed by both Flu and HCoV (11.9%, 5/42). The combinations of Flu/HAdV, Flu/HRV, and HAdV/MP each accounted for 7.1% (3/42) of co-infections (Figure 5). By individual pathogen, Flu (59.6%, 25/42) was the most frequently involved in co-infections, followed by MP (33.3%, 14/42), HRV (19.0%, 8/42), and HAdV (16.7%, 7/42). Age distribution analysis showed that co-infection was most common in the <18 years group (22.7%, 25/110, *p* = 0.012). Within pediatric subgroups, the highest co-infection rate was observed in children aged 1–3 years (Supplementary Table S3). We further analyzed clinical symptoms and laboratory findings, including cough, sore throat, sputum production, diarrhea, nausea, vomiting, fatigue, dizziness/headache, myalgia, nasal congestion, rhinorrhea, dyspnea, as well as white blood cell and lymphocyte counts. Compared with single infections, patients with co-infections were associated with significantly higher frequencies of nasal congestion (*p* = 0.006) and rhinorrhea (*p* = 0.039), but lower frequencies of dizziness/headache (*p* < 0.001) and myalgia (*p* = 0.007). Similar patterns were observed in pathogen-specific comparisons for Flu, MP, SARS-CoV-2, HAdV, and HRV.

### 3.3 Seasonal characteristics

**Figure 6:**
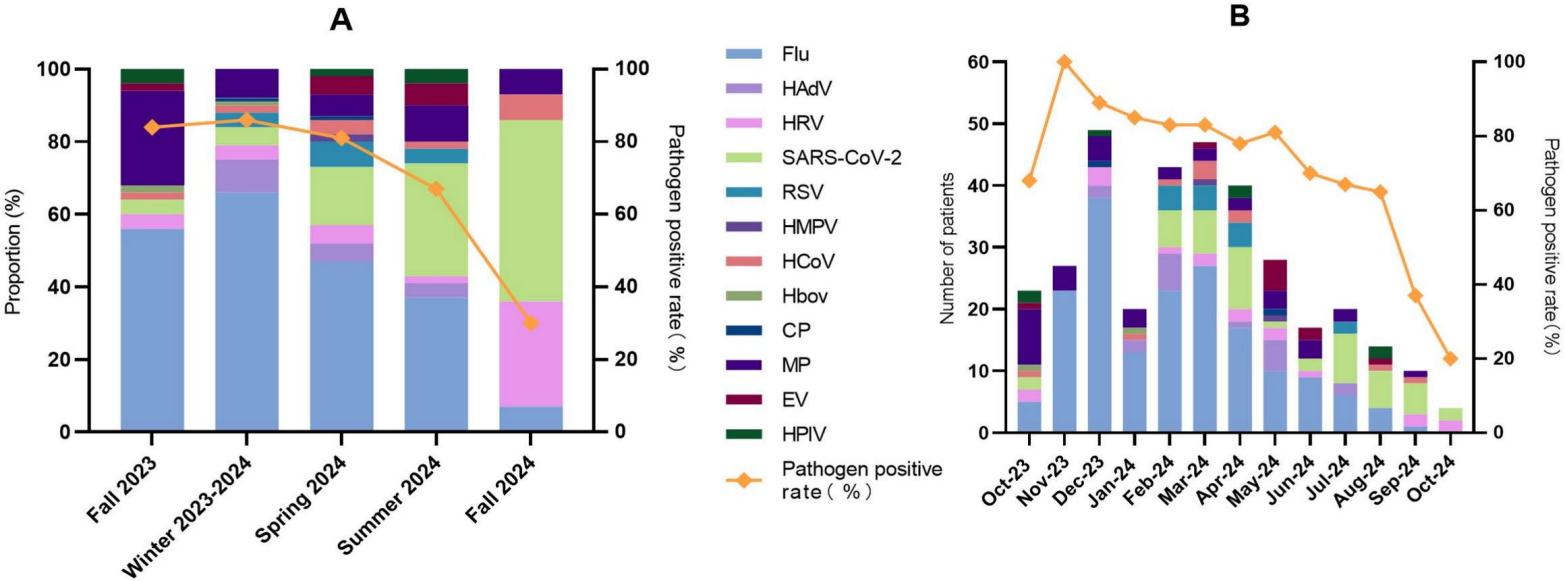
Monthly and seasonal patterns of respiratory pathogen detection. **(A)** Seasonal composition of individual respiratory pathogens. **(B)** Monthly trend of the overall pathogen-positive rate from October 2023 to October 2024.

Pathogen detection exhibited significant seasonal (*p* < 0.001) and monthly (*p* < 0.001) variation. Seasonally, detection rate was highest in the winter of 2023–2024 (85.7%, 96/112) and the autumn of 2023 (83.7%, 41/49) (Figure 6A). The pathogen spectrum showed successive dominance: Flu in autumn-winter of 2023–2024; Flu/SARS-CoV-2 co-circulation in spring–summer of 2024; and SARS-CoV-2/HRV dominance by autumn 2024 (Figure 6A; Supplementary Table S4). Monthly pathogen testing also showed the same trend. Consolidating the five original statistically homogeneous subsets, standardized residual analysis delineated three phases with significant inter-phase differences: high detection (Nov 2023-May 2024), moderate detection (Oct 2023, Jun-Aug 2024), and low detection (Sep-Oct 2024) (Supplementary Table S5). The positivity rate peaked in November 2023 (100%, 24/24), remained high through May 2024 (77.3–89.1%), and then declined (Figure 6B).

### 3.4 Influenza virus in Guilin, October 2023 to October 2024

Among the 400 samples, Flu was detected in 44.0% of cases (176/400). IBV demonstrated the highest detection rate at 17.5% (70/400), followed by H3N2 at 17.3% (69/400), while H1N1 showed the lowest rate of 9.3% (37/400). Among the 176 Flu-positive cases, 73 (41.5%) were male and 103 (58.5%) were female. The gender-specific distribution for each subtype was as follows: H1N1 (15 males, 22 females), H3N2 (32 males, 37 females), and IBV (26 males, 44 females) (*p* > 0.05 for all; Supplementary Table S6).

Detection rates of influenza subtypes varied across age groups. H1N1 showed peak detection in the 45–59 and ≥60 years groups (33.3% each), while H3N2 was most prevalent in the 45–59 years (20.8%) and 18–44 years (18.7%) groups. IBV predominated in the 18–44 years (20.5%) and <18 years (16.9%) groups. Age-specific differences were statistically significant for H1N1 (*p* < 0.001). In pediatric subgroups, H1N1 was most detected in <1 year (11.1%) and 1–3 years (7.1%) groups, H3N2 in 7–12 and 13–17 years groups (21.4% each), and IBV in 13–17 years (23.8%) and 4–6 years (17.1%) groups. None of these distributions reached statistical significance in the pediatric groups (*p* > 0.05; Supplementary Table S7).

Seasonal analysis revealed distinct peaks for each influenza subtype. H1N1 detection was highest in the summer 2024 (26.0%, 19/73), followed by the spring 2024 (14.3%, 17/119). H3N2 showed comparable peaks in the autumn 2023 (32.7%, 16/49) and the winter 2023–2024 (32.1%, 36/112). IBV was most prevalent in the winter 2023–2024 (33.9%, 38/112), then the autumn 2023 (24.5%, 12/49). All three subtypes exhibited statistically significant seasonal variations (*p* < 0.001; Supplementary Table S8).

**Figure 7:**
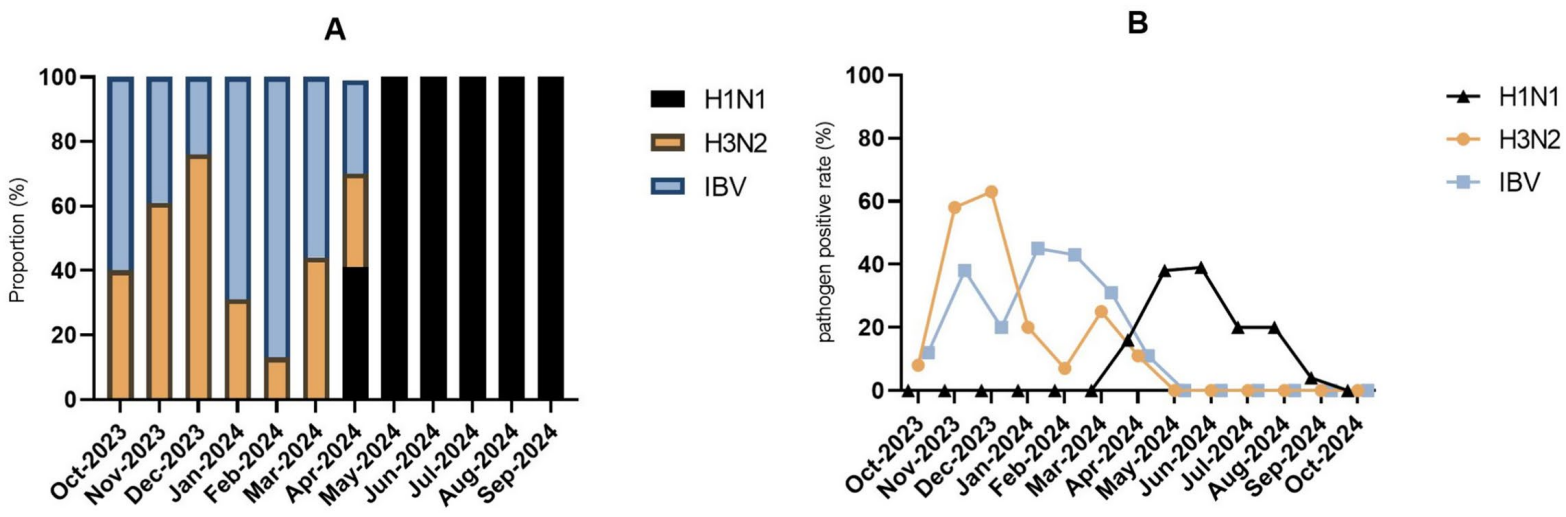
Prevalence and distribution of influenza virus subtypes in Guilin, China, from October 2023 to October 2024. **(A)** Monthly composition of three influenza viruses. **(B)** Monthly positive rate of three influenza viruses.

Monthly patterns further illustrated these dynamics. The three subtypes displayed distinct temporal distributions: during October 2023–March 2024, only H3N2 and IBV were detected; all three co-circulated in April 2024; thereafter, only H1N1 was detected from May to September 2024 (Figure 7A). Each subtype peaked during different months: H1N1 reached its highest rate in June 2024 (39.1%, 9/23), followed by May 2024 (38.5%, 10/26); H3N2 peaked in December 2023 (63.0%, 29/46), then November 2023 (58.3%, 14/24); IBV showed its highest detection in January 2024 (45.0%, 9/20), followed by February 2024 (43.5%, 20/46). All three subtypes exhibited statistically significant monthly variations (*p* < 0.001, Figure 7B).

## 4 Discussion

This study investigated the pathogen spectrum, epidemiological patterns, and population distribution of respiratory virus infections in Guilin by analyzing ILI patients from October 2023 to October 2024. Our findings revealed seasonal patterns, age-specific variations, and concurrent circulation of multiple pathogens in the region. These results provide important epidemiological evidence for prevention and treatment strategies for ARI/ILI in Guilin.

The overall detection rate of the 18 respiratory pathogens was up to 74.0% in this study, which was higher than the rates reported in similar pathogen spectrum studies from other regions in China during 2021–2024 (13–16). This high prevalence indicates that the vast majority of ILI cases in this study were attributable to known common pathogens. The detection of as many as 18 pathogens is also an important reason for the high positive rate in this study. Flu was the predominant pathogen (44.0%), significantly higher than that of other pathogens, and confirming it as the primary cause of ILI in Guilin during the study period. SARS-CoV-2 ranked second (12.3%), highlighting its transition from a pandemic pathogen to an established respiratory pathogen that co-circulates with other respiratory pathogens. Notably, MP and HAdV also showed considerable prevalence (8.8 and 4.5%, respectively), particularly among pediatric patients. This pattern underscores the ongoing roles of schools and childcare facilities as high-risk environments for the transmission of these pathogens (17). The simultaneous detection of 18 pathogens, mainly viruses, further emphasizes the complex and multi-pathogen nature of respiratory infections in this region. Regarding demographic distribution, no significant difference in pathogen detection was observed between genders. However, pathogen spectrum varied markedly across age groups. Differences in the detection rates of Flu, SARS-CoV-2, MP, HAdV, RSV, and others indicate the existence of age-specific susceptible populations for each pathogen. MP, HAdV, RSV, EV, and HPIV all showed the highest detection rates in the <18 years group, which is consistent with previous reports (18–22) and likely attributable to the immature immune system in children (23). Notably, the 45–59 years group exhibited the highest detection rates for both Flu (58.3%) and SARS-CoV-2 (29.2%), as well as the highest overall pathogen positivity (91.7%). This pattern may be explained by two factors: first, middle-aged adults (45–59 years) have high exposure risks due to their active social and professional engagement (24); second, this group may face an “immunity gap”—waning immunity from prior infections or vaccinations is not adequately boosted by follow-up immunizations, resulting in suboptimal protection compared to the older adults (who often have more consistent vaccination coverage) (25). This immunological vulnerability in middle-aged adults aligns with recent global influenza surveillance data showing increased case proportions in this age group (26, 27). However, due to the limited sample size of individuals aged >45 years in this study, these findings warrant further validation in larger population-based studies.

Further stratification of the pediatric patients revealed notably higher overall pathogen detection in children aged >1 year compared to infants under 1 year (44.4%). This difference may reflect limited environmental exposure during infancy, whereas entry into collective childcare settings (e.g., nurseries, kindergartens) increases exposure opportunities for children aged >1 year. The high prevalence of HAdV and RSV in young children is consistent with the established epidemiological profile of these pathogens, which are known to cause severe lower respiratory tract infections in this age group (4, 28). Specifically, the peak RSV detection in the 1–3 years subgroup (21.4%) aligns with recent findings from Wuxi, China, which confirmed the highest susceptibility to RSV among children under 3 years (29).

This study delineated the dynamic patterns of respiratory pathogens in Guilin. The pathogen detection rate peaked during autumn–winter 2023–2024 (November–February), followed by a gradual decline, aligning with the typical winter–spring epidemic pattern in southern China (28). Notably, we observed both succession and co-circulation of pathogens: Flu dominated alongside HAdV and MP from autumn to winter 2023, its prevalence subsequently waned in spring–summer 2024, accompanied by increasing proportions of SARS-CoV-2 and HRV, and by autumn 2024, SARS-CoV-2 and HRV had emerged as the predominant pathogens. This observed “pathogen succession” represents a common feature of respiratory virus ecology, which may be driven by fluctuations in population immunity, climatic conditions, and behavioral factors (30, 31). The epidemic trends of Flu and SARS-CoV-2 were consistent with national ARI surveillance data during the same period (32). Specifically, SARS-CoV-2 detection rates in Guilin increased during July–September 2024, peaking in August (30.0%) before declining to 10.0% in October. This trend closely mirrored national sentinel surveillance data, which reported SARS-CoV-2 positivity rising from 5.4% in May to 21.1% in August, then decreasing to 4.1% in October. The concordance between our local findings and national data further validates the representativeness of our surveillance and underscores the synchronized circulation of SARS-CoV-2 across broader regions during this period.

An in-depth analysis of influenza subtypes revealed a complex epidemiological pattern. Distinct subtype dominance was observed across the study period: IBV and H3N2 co-circulated during the autumn-winter of 2023–2024, while H1N1 emerged later and peaked in spring–summer 2024. Notably, all three subtypes co-circulated in April 2024, after which H1N1 became the only detected subtype from May to September. This sequential subtype replacement aligns with national influenza surveillance data (33, 34), underscoring the importance of precise monitoring for guiding vaccine strain selection (35). Age-specific patterns were also evident for Flu subtypes: H1N1 detection rates were higher in middle-aged and older adults (36). H3N2 was most prevalent among young and middle-aged adults (18–59 years), consistent with previous reports that this subtype accounts for 47.9% of Flu infections in this age group (37), and IBV showed predilection for children and young adults, which aligns with its established tendency to affect younger populations (38, 39). These findings are further supported by a 2023 study, which reported median ages of 59, 34.1, and 19.3 years for H1N1, H3N2, and IBV infections, respectively (*p* < 0.01) (40).

The co-infection rate of 14.2% was observed in this study, which is consistent with previous surveillance reports from Guilin (41). The most common co-infection patterns were Flu/MP and Flu/HCoV. Notably, a study from Henan identified IAV/IBV as the most common co-infection pattern during 2015–2023 (42); however, this combination was not detected in our cohort, suggesting substantial regional and temporal variations in the co-circulation of respiratory pathogens. Age distribution analysis revealed that co-infection was most common in the 0–17 years pediatric group (22.7%, 25/110), significantly higher than in other age groups (*p* = 0.015). This finding indicates that children are not only at high risk for respiratory pathogen infection but also represent a key population for co-infection. Among pediatric subgroups, the highest co-infection rate was observed in children aged 1–3 years, and although the difference across subgroups was not statistically significant, its clinical and epidemiological relevance warrants attention. The high frequency of co-infection in children may be attributed to their immature immune systems and frequent exposure in group settings such as daycare centers, which facilitate pathogen exposure and cross-transmission. These findings suggest that co-infection should be considered in the differential diagnosis of pediatric respiratory illness during epidemic seasons.

We further compared the clinical symptom profiles between co-infected and mono-infected patients. Co-infected patients exhibited significantly higher frequencies of upper respiratory symptoms (nasal congestion and rhinorrhea), but significantly lower frequencies of systemic symptoms (dizziness/headache and myalgia). This pattern was consistently observed across pathogen-specific comparisons for Flu, MP, SARS-CoV-2, HAdV, and HRV-likely attributable to the high representation of these pathogens in co-infection events. As all patients in this study were outpatients with mild illness, we lacked data on hospitalization, severe outcomes, or intensive care, and the sample sizes for certain virus-specific co-infection subgroups were limited, precluding precise assessment of the association between co-infection and disease severity. Nevertheless, previous studies have reported inconsistent findings. For instance, a study of hospitalized children with MP and other respiratory virus co-infections reported that MP co-infections may lead to more severe clinical manifestations, including prolonged fever, increased risk of pneumonia, and higher hospitalization rates (43). Conversely, another study found no significant difference in disease severity between mono-infected and co-infected patients with H1N1, HRV, RSV, HCoV, or HBoV. Additionally, some evidence suggests that HRV infection may reduce the risk of H1N1 infection (44). These findings indicate that co-infection does not invariably equate to severe illness; rather, its clinical impact may depend on specific pathogen combinations. Although the present study did not directly assess disease severity, the substantial prevalence of co-infections observed (14.2%) still underscores the clinical and public health value of implementing comprehensive multi-pathogen testing during periods of high respiratory virus activity. Even in the absence of severe systemic symptoms, accurate etiological diagnosis can inform clinical decision-making, support antimicrobial stewardship, and enhance respiratory pathogen surveillance systems.

In conclusion, this study confirms influenza virus as the predominant pathogen causing ILI in Guilin from October 2023 to October 2024, co-circulating with other pathogens including SARS-CoV-2 and MP, and exhibiting distinct seasonal patterns and demographic distributions. Molecular epidemiological and genetic evolutionary analyses are currently underway. However, the study has several limitations: First, limited sample size, uneven monthly collection, and age imbalance may affect the precision of temporal trends and age-specific estimates. Second, all patients were outpatients with mild symptoms, limiting generalizability to severe or hospitalized cases; the lack of systematic severity data and follow-up information precluded robust assessment of the association between infection (particularly co-infection) and disease severity. Third, individual-level vaccination history was unavailable, preventing evaluation of vaccine impact on detection rates and clinical outcomes. Despite these limitations, this study provides critical baseline epidemiological data for Guilin and southern China, identifying high-burden populations, including middle-aged adults for influenza, and young children for MP, HAdV, EV, and RSV—to inform future vaccine effectiveness evaluations and targeted immunization strategies. Future surveillance should integrate outpatient and inpatient cohorts, incorporate standardized severity assessments, individual vaccination records and longitudinal follow-up, and adopt larger-scale, balanced sampling to comprehensively characterize respiratory pathogen epidemiology and assess real-world vaccine effectiveness.

## Data Availability

The original contributions presented in the study are included in the article/Supplementary material, further inquiries can be directed to the corresponding authors.
