# Supplementary material for: The pathogen spectrum of influenza-like illness in Guilin, China, 2023–2024
Source: Front Public Health. 2026 Mar 18;14:1788314. doi: 10.3389/fpubh.2026.1788314 (PMC13054191; doi:10.3389/fpubh.2026.1788314)
Supplement: Supplementary file 1 [file Supplementary_file_1.docx]

Supplementary Material

# Supplementary Figures and Tables

**Supplementary Table S1** Detection of respiratory pathogens by gender.

|  | Male(n=187) | | Female(n=213) | | χ² | *P*-value |
| --- | --- | --- | --- | --- | --- | --- |
|  | Positive, n (%) | Negative, n (%) | Positive, n (%) | Negative, n (%) |  |  |
| Total samples | 131(70.1) | 56(29.9) | 165(77.5) | 48(22.5) | 2.843 | 0.092 |
| Flu | 73(39.0) | 114(61.0) | 103(48.4) | 110(51.6) | 3.51 | 0.061 |
| SARS-CoV-2 | 22(11.8) | 165(88.2) | 27(12.7) | 186(87.3) | 0.77 | 0.781 |
| MP | 15(8.0) | 172(92.0) | 20(9.4) | 193(90.6) | 0.233 | 0.629 |
| HAdV | 9(4.8) | 178(95.2) | 9(4.2) | 204(95.8) | 0.8 | 0.777 |
| HRV | 10(5.3) | 177(94.7) | 7(3.3) | 206(96.7) | 1.04 | 0.308 |
| RSV | 5(2.7) | 182(97.3) | 9(4.2) | 204(95.8) | 0.71 | 0.411 |
| EV | 5(2.7) | 182(97.3) | 5(2.3) | 208(97.7) | 0.044 | 0.835 |
| HCoV | 8(4.3) | 179(95.7) | 2(0.9) | 211(99.1) |  | 0.050* |
| HPIV | 4(2.1) | 183(97.9) | 3(1.4) | 210(98.6) |  | 0.715* |
| HMPV | 0(0.0) | 187(100.0) | 2(0.9) | 211(99.1) |  | 0.501* |
| HBoV | 0(0.0) | 187(100.0) | 2(0.9) | 211(99.1) |  | 0.501* |
| CP | 0(0.0) | 187(100.0) | 2(0.9) | 211(99.1) |  | 0.501* |

Data are presented as n (%). P-values were calculated by the Chi-square test (* Fisher's exact test). A P-value < 0.05 was considered statistically significant and is highlighted in **bold**.

**Supplementary Table S2** Detection of respiratory pathogens by age group.

| Age(years) | Number of samples, n (%) | | | *P*-value |
| --- | --- | --- | --- | --- |
|  | Total samples (N=400) | Positive (n=296) | Negative (n=104) |  |
| <18 | 142 | 110(77.5) | 32(22.5) | 0.072 |
| 18-44 | 219 | 154(70.3) | 65(29.7) |  |
| 45-59 | 24 | 22(91.7) | 2(8.3) |  |
| >60 | 15 | 10(66.7) | 5(33.3) |  |
| <1 | 9 | 4(44.4) | 5(55.6) | **0.015** |
| 1-3 | 28 | 22(78.6) | 6(21.4) |  |
| 4-6 | 35 | 32(91.4) | 3(8.6) |  |
| 7-12 | 28 | 24(85.7) | 4(14.3) |  |
| 13-17 | 42 | 28(66.7) | 14(33.3) |  |

Data are presented as n (%). P-values were calculated by the Chi-square test (Fisher-Freeman-Halton). A P-value < 0.05 was considered statistically significant and is highlighted in **bold**.

**Supplementary Table S3** Age distribution of co-infection and single infection cases.

| **Age (years)** | **Total positive, N** | **Co-infection, n (%)** | **Single infection, n (%)** | ***P-value*** |
| --- | --- | --- | --- | --- |
| <18 | 110 | 25 (22.7) | 85 (77.3) | **0.012** |
| 18-44 | 154 | 14 (9.1) | 140 (90.9) |  |
| 45-59 | 22 | 2 (9.1) | 20 (90.9) |  |
| >60 | 10 | 1 (10.0) | 9 (90.0) |  |
| <1 | 4 | 0 (0.0) | 4 (100) | 0.521 |
| 1-3 | 22 | 7 (31.8) | 15 (68.2) |  |
| 4-6 | 32 | 6 (18.8) | 26 (81.3) |  |
| 7-12 | 24 | 7 (29.2) | 17 (70.8) |  |
| 13-17 | 28 | 5 (17.9) | 23 (82.1) |  |

Data are presented as n (%). P-values were calculated by the Chi-square test (Fisher-Freeman-Halton). A P-value < 0.05 was considered statistically significant and is highlighted in **bold**.

**Supplementary Table S4** Detection counts of respiratory pathogens by season.

|  | Fall 2023 | Winter 2023-2024 | Spring 2024 | Summer 2024 | Fall 2024 | Total |
| --- | --- | --- | --- | --- | --- | --- |
| Number of samples | 49 | 112 | 119 | 73 | 47 | 400 |
| Total positive samples, n (%)^￥^ | 41 (83.7) | 96 (85.7） | 96 (80.7） | 49 (67.1） | 14 (9.8) | 296^#^ (74.0%) |
| Flu^*^ | 28 (57.1) | 74 (66.1) | 54 (47.0) | 19 (26.0) | 1 (2.1) | 176 (44.0) |
| HAdV^*^ | 0 (0.0) | 10 (8.9) | 6 (5.0) | 2 (2.7) | 0 (0.0) | 18 (4.5) |
| HRV^*^ | 2 (4.0) | 4 (3.6) | 6 (5.0) | 1 (1.4) | 4 (8.5) | 17 (4.3) |
| SARS-CoV-2^*^ | 2 (4.0) | 6 (5.4) | 18 (15.1) | 16 (21.9) | 7 (14.9) | 49 (12.3) |
| RSV^*^ | 0 (0.0) | 4 (3.6) | 8 (6.7) | 2 (2.7) | 0 (0.0) | 14 (3.5) |
| HMPV^*^ | 0 (0.0) | 0 (0.0) | 2 (1.7) | 0 (0.0) | 0 (0.0) | 2 (0.5) |
| HCoV^*^ | 1 (2.0) | 2 (1.8) | 5 (4.2) | 1 (1.4) | 1 (2.1) | 10 (2.5) |
| Hbov^*^ | 1 (2.0) | 1 (0.9) | 0 (0.0) | 0 (0.0) | 0 (0.0) | 2 (0.5) |
| CP^*^ | 0 (0.0) | 1 (0.9) | 1 (0.8) | 0 (0.0) | 0 (0.0) | 2 (0.5) |
| MP^*^ | 13 (26.5) | 9 (8.0) | 7 (5.9) | 5 (6.9) | 1 (2.1) | 35 (8.8) |
| EV^*^ | 1 (2.0) | 0 (0.0) | 6 (5.0) | 3 (4.1) | 0 (0.0) | 10 (2.5) |
| HPIV^*^ | 2 (4.1) | 1 (0.9) | 2 (1.7) | 2 (2.7) | 0 (0.0) | 7 (1.8) |

^￥^Data are presented as n (%). Percentages reflect the sample-positive rate.

^*^Data are number of detections (%). Percentages are based on the total pathogen detection events, not on the number of positive samples.

^#^The total number of detections exceeds the number of positive samples due to co-infections.

**Supplementary Table S5** Statistically homogeneous subsets of monthly detection rates.

| Phase | Key Characteristics | Subset Letters |  | Months Included | Detection Range (%) | Mean Detection (%) |
| --- | --- | --- | --- | --- | --- | --- |
| High-Detection | Sustained high transmission period with no significant within-phase differences | c |  | Nov 2023, Dec 2023, Feb 2024, Mar 2024, Apr 2024 | 77.8-100.0 | 86.6 |
|  |  | b, c |  | Jan 2024, May 2024 | 80.8-85.0 | 82.9 |
| Moderate-Detection | Stable intermediate transmission levels | a, b, c |  | Oct 2023, Jun 2024, Jul 2024, Aug 2024 | 65.0-69.6 | 67.3 |
| Low-Detection | Significant epidemiological downturn | a, b |  | Sep-24 | 37 | 37 |
|  |  | a |  | Oct-24 | 20 | 20 |

1. Subsets were identified based on standardized residual analysis with Bonferroni correction (α=0.05).
2. Months sharing the same letter within each detection category constitute statistically homogeneous subsets (p>0.05), whereas months with different letters show significant differences in detection rates (p<0.05).
3. Months within the same phase show no significant differences in detection rates (p>0.05).
4. Detection rates differ significantly between phases: High vs. Low phases, p<0.001; High vs. Moderate phases, p<0.01.

**Supplementary Table S6** Detection of influenza virus subtypes by gender in the study population (N=400).

|  | **Total, n (%)** | **Male, n (%)** | **Female, n (%)** | **χ² value** | ***P*-value** |
| --- | --- | --- | --- | --- | --- |
| **Influenza Virus Subtype** | 176 (44.0) | 73 (41.5) | 103 (58.5) | 3.51 | 0.061 |
| H1N1 | 37 (9.3) | 15 (40.5) | 22 (59.5) | 0.017 | 0.896 |
| H3N2 | 69 (17.3) | 32 (46.4) | 37 (53.6) | 1.122 | 0.289 |
| IBV | 70 (17.5) | 26 (37.1) | 44 (62.9) | 0.9 | 0.343 |

Data are presented as n (%). P-values were calculated by the Chi-square test. A P-value < 0.05 was considered statistically significant and is highlighted in **bold**.

**Supplementary Table S7** Detection of influenza viruses across different age groups.

**Part A: Total Study Population (N=400)**

| **Age Group** | **Total sample** | **H1N1, n (%)** | **H3N2, n (%)** | **IBV, n (%)** | **Influenza-positive, n (%)** |
| --- | --- | --- | --- | --- | --- |
| 0-17 yr | 142 | 4 (2.8) | 20 (14.1) | 24 (16.9) | 48 (33.8) |
| 18-44 yr | 219 | 20 (9.1) | 42 (19.2) | 45 (20.5) | 107 (48.9) |
| 45-59 yr | 24 | 8 (33.3) | 5 (20.8) | 1 (4.2) | 14 (58.3) |
| ≥60 yr | 15 | 5 (33.3) | 2 (13.3) | 0 (0.0) | 7 (46.7) |
| **χ² value** |  |  |  |  | **10.135** |
| ***P*-value** |  | **<0.001*** | 0.642* | 0.062* | **0.017** |

**Part B: Pediatric Sub-cohort (n=142)**

| **Age Group** | **Total sample** | **H1N1, n (%)** | **H3N2, n (%)** | **IBV, n (%)** | **Influenza-positive, n (%)** |
| --- | --- | --- | --- | --- | --- |
| <1 yr | 9 | 1 (11.1) | 0 (0.0) | 1 (11.1) | 2 (22.2) |
| 1-3 yr | 28 | 2 (7.1) | 2 (7.1) | 4 (14.3) | 8 (28.6) |
| 4-6 yr | 35 | 0 (0.0) | 3 (8.6) | 6 (17.1) | 9 (25.7) |
| 7-12 yr | 28 | 0 (0.0) | 6 (21.4) | 4 (14.3) | 10 (35.7) |
| 13-17 yr | 42 | 1 (2.4) | 9 (21.4) | 10 (23.8) | 20 (47.6) |
| ***P*-value** |  | 0.251* | 0.210* | 0.732* | 0.284* |

Data are presented as n (%). P-values were calculated by the Chi-square test (* Fisher-Freeman-Halton). A P-value < 0.05 was considered statistically significant and is highlighted in **bold**.

**Supplementary Table S8** Seasonal detection of influenza virus subtypes.

| **Season** | **Total samples**  **（N=400）** | **H1N1, n (%)** | **H3N2, n (%)** | **IBV, n (%)** | Influenza-positive, n (%) |
| --- | --- | --- | --- | --- | --- |
| **Fall 2023** | 49 | 16 (32.7) | 12 (24.5) | 0 (0.0) | 28 (57.1) |
| **Winter 2023-2024** | 112 | 0 (0.0) | 36 (32.1) | 38 (33.9) | 74 (66.1) |
| **Spring 2024** | 119 | 17 (14.3) | 17 (14.3) | 20 (16.8) | 54 (45.4) |
| **Summer 2024** | 73 | 19 (26.0) | 0 (0.0) | 0 (0.0) | 19 (26.0) |
| **Fall 2024** | 47 | 1 (2.1) | 0 (0.0) | 0 (0.0) | 1 (2.1) |
| ***P*-value** |  | **<0.001** | **<0.001** | **<0.001** | **<0.001** |

Data are presented as n (%). P-values were calculated by the Chi-square test (Fisher-Freeman-Halton). A P-value < 0.05 was considered statistically significant and is highlighted in **bold**.
